# Prognostic risk factors for early outcomes of patients with myelomeningocele: a prospective study

**DOI:** 10.1007/s00381-024-06455-z

**Published:** 2024-06-07

**Authors:** Seyed Ahmad Naseri Alavi, Amir Rezkhah, Alireza Majdi, Mohammad Amin Habibi, Mohammad Mehdi Bagheri, Fateme Jafarzadeh, Andrew J. Kobets

**Affiliations:** 1 Department of Neurosurgery, School of Medicine, Emory University, Atlanta, GA USA; 2 Department of Neurosurgery, Urmia University of Medical Sciences, Urmia, Iran; 3 Research Group Experimental Oto-rhino-laryngology, Department of Neuroscience, Leuven Brain Institute, KU Leuven, Leuven, 3000 Belgium; 4 Department of Neurosurgery, Shariati Hospital, Tehran University of Medical Sciences, Tehran, Iran; 5 Department of Neurological Surgery, Montefiore Medical, Bronx, NY USA

**Keywords:** Neural tube defects, Myelomeningocele, Risk factors, Early outcomes

## Abstract

**Introduction:**

Myelomeningocele (MMC) is a prevalent form of neural tube defect. Despite advancements in treatment, MMC still poses significant health risks, including complications leading to chronic disability and mortality. Identifying prognostic risk factors for early outcomes is crucial for tailored intervention strategies.

**Methods:**

This prospective study involved newborns and infants diagnosed with MMC who underwent surgery between 2020 and 2023 at Urmia University of Medical Sciences. Demographic data and surgical outcomes were collected, and participants were followed up for six months. Statistical analyses were conducted using descriptive statistics, Chi-Square, and independent t-test.

**Results:**

The study included 29 MMC cases, with an incidence rate of 1.4 per 10,000 live births. Lesions were predominantly located in the lumbar spine. Although mortality rates appeared to increase with ascending lesion sites, this trend was not statistically significant. Short-term outcomes revealed high morbidity and mortality rates, with neurological deficits being the most prevalent complication. Multivariable analysis identified head circumference as a significant predictor of adverse outcomes (IRR = 1.37, 95% CI = 1.02 to 1.86, *p* = 0.04). Furthermore, an increase in birth weight was associated with a reduction in the incidence of requiring a ventriculoperitoneal shunt (IRR = 0.99, 95% CI = 0.998 to 0.999, *p* = 0.02).

**Conclusion:**

This prospective study highlights prognostic risk factors for early outcomes in MMC patients, emphasizing the need for personalized intervention strategies. By addressing modifiable risk factors and implementing targeted interventions, healthcare providers can strive to improve outcomes and enhance the quality of life for MMC patients.

## Introduction

Neural tube defects (NTDs) manifest in 0.5–8 per 1000 live births, constituting congenital anomalies that impact the central nervous system (CNS) [1]. Myelomeningocele (MMC) stands as the prevailing form of NTD, distinguished by a dorsal midline lesion comprising a neural plaque (placode) attached to neighboring dysplastic epithelial tissue or a cystic lesion characterized by a herniated sac that protrudes through a defect in the vertebral bone, encompassing the meninges, spinal cord, and cerebrospinal fluid (CSF) [2]. MMC represents not merely a congenital anomaly but also poses significant health risks, including complications such as hydrocephalus, neurogenic bladder, Chiari malformation type II (CM-II), motor impairments, and orthopedic abnormalities. These complications have been identified as significant contributors to patient morbidity and mortality [3, 4].

In the past, the survival rate for individuals diagnosed with MMC was below 10% [3]. Recent advancements in medical treatment have led to a notable increase in the number of children surviving MMC and reaching adulthood. However, despite these improvements, MMC remains challenging as it can result in chronic disability [4–6].

Identifying cases of MMC at higher risk of developing complications early on presents a significant challenge in postnatal management [7, 8]. In a study by Kaufman et al. [7], the inadequate follow-up of these patients was linked with heightened morbidity. In that light, prompt recognition of such cases allows for the implementation of tailored treatment approaches. Therefore, it is imperative to comprehend the involved prognostic factors in this context.

This study endeavors to pinpoint the risk factors that may contribute to sudden death among adolescent and young adult patients. By doing so, we aim to diminish the death incidence and enhance the health and well-being of patients suffering from MMC. Through a comprehensive analysis, we examine the diverse factors influencing morbidity and mortality rates in individuals with MMC. This allows us to identify those at risk and implement appropriate preventive measures to avert such events.

## Participants and methods

This study adhered to the STROBE guideline for epidemiological observational studies and was conducted within the approved ethical framework by the ethics committee of Urmia University of Medical Sciences.

### Inclusion and exclusion criteria

This prospective study enrolled infants diagnosed with MMC whose guardians/parents provided written informed consent. These infants were referred to Urmia University of Medical Sciences for MMC repair surgery between 2020 and 2023. Subsequently, participants were prospectively followed up for a period of six months. Inclusion criteria comprised maternal age between 18 and 35 years, regardless of gestational week, normal karyotype, MMC lesion at the level of S1 or higher, and active participation in follow-up assessments. Exclusion criteria included the presence of chronic conditions such as insulin-dependent gestational diabetes or obesity with a BMI ≥35, multiple gestational pregnancies, history of previous abortion, additional fetal anomalies, placenta previa, incompetent cervix or short cervix <20 mm, fetal kyphosis ≥30 degrees, maternal-fetal Rh immunization, positive maternal human immunodeficiency (HIV) or hepatitis-B (HBV) or hepatitis-C (HCV) viruses infection, uterine anomaly, and inability to comply with follow-up appointments.

### Data collection

Demographic data, including age, sex, gestational week, birth weight, and head circumference (HC), as well as information on infant comorbidities and Apgar scores at 1 minute and 5 minutes post-birth were gathered. Additionally, details regarding the delivery method and surgical outcomes such as the need for a ventriculoperitoneal shunt (VPS), wound infection, CNS infection, neurological deficit, movement deficit, and urinary deficit were recorded. All patients were followed up for six months to assess their outcomes.

### Study outcomes

The primary outcome of the study was to assess the morbidity and mortality rates associated with MMC repair surgery in patients. Secondary outcomes included evaluating short-term complications such as the need for a VPS, wound infection, CNS infection, and neurological deficits e.g., movement, and urinary deficits following the surgery.

### Statistical analyses

The data analysis employed descriptive statistics tests, presenting results as mean ± standard deviation (SD) for quantitative variables and frequency as a percentage for qualitative variables. Qualitative variables were assessed using the Chi-Square test, while the independent t-test was applied to compare quantitative variables. IBM SPSS Statistics for Windows, version 28 (IBM Corp., Armonk, NY, USA), was employed for data analysis, with statistical significance set at *p* < 0.05.

## Results

### General findings

Between March 2019 and February 2023, there were 212,077 new births in West Azerbaijan province, among which 29 cases were diagnosed with MMC, resulting in an incidence rate of 1.4 per 10,000 live births. The baseline characteristics of the newborns are presented in Table 1. The median age at surgery was seven days (IQR = 3.5 to 17.5), with a minimum of two and a maximum of 205, indicating a positive skewness in the data (Skewness = 2.35). Although the median age at surgery was higher in the mortality-positive group, the difference was not statistically significant (medians = 7.5 vs. 6). The mean lesion diameter was found to be 5.58 ± 2.1 cm, ranging from 2 to 10 cm.

**Table 1:** The baseline characteristics of the newborns

**Characteristic**	**Mortality**		**Overall**
	** Yes **	** No **	
Sex			
Male	7(77.8%)	2(22.2%)	9(31%)
Female	14(70%)	6(30%)	20(69%)
Gestation age (w)	37.25(3.41)	38.05(1.2)	37.83(2.02)
Birth weight (gr)	3100(700.51)	3219.1(799.61)	3186(763)
Head circumference (cm)	35.25(3.28)	33.14(2.71)	33.72(2.98)
Apgar at 1 minute	6.88(1.81)	7.76(1.48)	7.52(1.60)
Apgar at 5 minute	8.25(1.49)	9.38(0.87)	9.07(1.16)
Delivery method			
NVD	4(40%)	6(60%)	10(34.5%)
C/S	15(78.9%)	4(21.1%)	19(65.5%)

*NVD* natural vaginal delivery, *C/S* cesarian section

### Anatomical level

The majority of MMC cases were located in the lumbar spine (75.9%), followed by the thoracic spine (17.2%). Only two patients had MMC at the cervical spine. Although we observed a trend suggesting an increase in the mortality rate corresponding to the ascending lesion site in the spinal cord (Table 2), this association did not reach statistical significance (*p* = 0.17).

**Table 2:** Anatomical site of the lesion in the newborn

**Lesion site** **(Spine)**	**Mortality**	
	** Yes **	** No **
Cervical	1(50%)	1(50%)
Thoracic	2(40%)	3(60%)
Lumbar	9(22.73%)	17(77.27%)

### Primary and secondary outcomes

The short-term outcomes of participants are outlined in Table 3. The overall 6-month mortality rate among participants was 27.6%. Only one case was free from complications, while 96.6% experienced at least one complication, with the neurological deficit being the most prevalent one (65.5%).

**Table 3:** Primary and secondary outcomes in the included prospective cohort

**Outcomes**	**Frequency (%)**
Any complications	28(96.6%)
Ventriculoperitoneal shunt	15(51.7%)
Wound infection	13(44.8%)
Central nervous system infection	9(31%)
Neurological deficit	19(65.5%)
Movement deficit	18(62.1%)
Urination deficit	0
Death	8(27.6%)

Using multivariable analysis, it was found that a one-unit change in HC may increase the shunt incidence rate by 25.7% (IRR=1.26, 95% CI = 0.98 to 1.62, *p* = 0.07). Change in HC was also found to be associated with an increased incidence rate of meningitis (IRR=1.37, 95% CI = 1.02 to 1.86, *p* = 0.04) and surgical site infection (IRR=1.25, 95% CI = 1.002 to 1.56, *p* = 0.04). Additionally, a 100-gram increase in birth weight was associated with a 13% reduction in the incidence rate of shunting (IRR = 0.99, 95% CI = 0.998 to 0.999, *p* = 0.02) (Table 4).

**Table 4:** The correlation between study variables and primary and secondary outcomes at 6 months old

Outcomes	*P* value	IRR (95% CI)
**6-month mortality**		
Gestation age (w)	0.36	0.89(0.69 to 1.15)
Birth weight (gr)	0.58	1(0.99 to 1)
Head circumference (cm)	0.34	1.15 (0.86 to 1.53)
Apgar at 5 minute	0.23	0.69 (0.37 to 1.27)
**Ventriculoperitoneal shunt**		
Gestation age (w)	0.86	1.02 (0.78 to 1.34)
Birth weight (gr)	0.02*	0.99 (0.998 to 0.999)
Head circumference (cm)	0.07*	1.26 (0.98 to 1.62)
Apgar at 5 minute	0.92	0.97 (0.61 to 1.57)
**Wound infection**		
Gestation age (w)	0.89	0.98 (0.74 to 1.3)
Birth weight (gr)	0.62	0.99 (0.998 to 1)
Head circumference (cm)	0.04*	1.25 (1 to 1.56)
Apgar at 5 minute	0.93	1.02 (0.62 to 1.68)
**CNS infection**		
Gestation age (w)	0.33	0.87 (0.65 to 1.15)
Birth weight (gr)	0.59	0.99 (0.998 to 1)
Head circumference (cm)	0.04*	1.37 (1.02 to 1.86)
Apgar at 5 minute	0.99	1 (0.54 to 1.88)
**Neurological deficits**		
Gestation age (w)	0.78	0.97 (0.79 to 1.2)
Birth weight (gr)	0.45	0.99 (0.99 to 1)
Head circumference (cm)	0.75	1.03 (0.86 to 1.23)
Apgar at 5 minute	0.4	0.84 (0.56 to 1.26)
**Movement deficits**		
Gestation age (w)	0.91	0.99 (0.77 to 1.26)
Birth weight (gr)	0.84	0.99 (0.999 to 1)
Head circumference (cm)	0.44	0.93 (0.76 to 1.13)
Apgar at 5 minute	0.83	1.05 (0.66 to 1.68)

*IRR* incidence ratio rate, *CNS* central nervous system

*Denotes a significant correlation between the assessed variables

## Discussion

MMC remains a significant congenital anomaly affecting the CNS, often leading to severe health complications and mortality in affected individuals [8]. This prospective study aimed to identify prognostic risk factors associated with early outcomes in patients with MMC. Our findings shed light on crucial factors influencing morbidity and mortality rates in this population, facilitating the development of tailored interventions to improve patient care and outcomes.

### Incidence and location

Consistent with previous literature [1, 8], our study revealed a relatively low incidence of MMC, with 1.4 cases per 10,000 live births in West Azerbaijan province. The majority of MMC cases were located in the lumbar spine, highlighting the typical anatomical presentation of this condition. Notably, while there was a trend suggesting an association between higher lesion sites in the spinal cord and increased mortality rates, this correlation did not reach statistical significance. Previous studies [9, 10] have shown that the location of the spinal defect holds clinical significance. Lesions affecting upper levels of the spinal cord and nerve roots tend to have a poorer neurological prognosis compared to lesions at lower levels. Over time, the level of spinal cord injury may impact academic, neurobehavioral, and intellectual functioning.

### Short-term outcomes and complications

Our study documented significant short-term morbidity and mortality among MMC patients, with a 27.6% mortality rate observed within six months of birth. In a study by Rodrigues et al. [10], the mortality rate in the studied group of patients was found to be 4.2%. The main causes of death in this group were aspiration pneumonia, sepsis, and CNS infection. Protzenko et al. [8], demonstrated seventeen deaths (7.4%) during the study period. shunt dysfunction, infection and proximal obstruction, urinary sepsis, and renal failure were the most common causes of mortality in this study. In our study, nearly all patients experienced at least one complication, with neurological deficit being the most prevalent outcome. These findings underscore the substantial burden of MMC on patient health and highlight the importance of early intervention and comprehensive management strategies to mitigate adverse outcomes.

### Prognostic factors and intervention strategies

Our multivariable analysis identified several prognostic risk factors associated with early outcomes in MMC patients. Notably, HC emerged as a significant predictor of adverse outcomes, with increases linked to higher incidences of meningitis and surgical site infections. Accordingly, it has been shown that neonates lacking antenatal hydrocephalus (and thus lower HC) had a 1.73-fold reduced likelihood of experiencing a combination of surgical wound complications, CNS infection, requirement for a ventricular shunt, and necessitating resuscitation in the delivery room [10]. Conversely, in a study involving 67 patients with neural tube defects who underwent a total of 122 shunt procedures, HC at the time of shunt insertion did not demonstrate a correlation with patient survival [11]. We also found that higher birth weight was associated with a reduced incidence of shunting, suggesting potential protective effects against certain complications. Considering all the aforementioned points, age at the time of surgery surfaced as a more dependable gauge of patient maturity and thus a better predictor of outcomes, owing to its robust association with weight and HC, especially given that HC may be influenced by the severity of hydrocephalus [12].

Therefore, it is logical to expect a higher mortality rate in populations with low birth weight at birth and early surgery, as we demonstrated in this study. Additionally, there might be other hidden factors, such as ICU care and post-operative recovery, which could be more significant contributors to the elevated mortality rate but have not been discussed [13, 14].

### Study limitations and future directions

While our study provides valuable insights into prognostic risk factors for early outcomes in MMC patients, several limitations warrant consideration. The relatively small sample size and single-center design may limit the generalizability of our findings. Future research efforts should aim to validate these results in larger, multicenter studies to enhance their applicability and robustness. Additionally, longitudinal follow-up beyond six months is needed to assess long-term outcomes and identify potential predictors of late complications in MMC patients.

## Conclusion

Our prospective study identifies prognostic risk factors associated with early outcomes in patients with MMC. These findings underscore the complex nature of MMC management and highlight the importance of comprehensive assessment and intervention strategies tailored to individual patient characteristics. By addressing modifiable risk factors and implementing targeted interventions, healthcare providers can strive to improve outcomes and enhance the quality of life for MMC patients.

## Data Availability

No datasets were generated or analysed during the current study.
